# How can the digital economy reduce carbon emissions? Empirical evidence from China

**DOI:** 10.1371/journal.pone.0303582

**Published:** 2024-06-25

**Authors:** Mingyue Chen, Xiaowen Wang, Zhenhua Zhang

**Affiliations:** School of Economics, Lanzhou University, Gansu, China; Second Xiangya Hospital, Central South University, CHINA

## Abstract

China is transitioning into the digital economy era. The advancement of the digital economy could offer a fresh mechanism to attain carbon peak and carbon neutrality objectives. Applications of the digital economy, such as smart energy management, intelligent transport systems, and digital agricultural technologies, have significantly reduced carbon emissions by optimizing resource use, reducing energy waste, and improving production efficiency. This research does so by devising a theoretical model that looks into the multi-faceted power of the digital economy under a two-sector paradigm. Utilising a panel model, a mediation effect model and a spatial Durbin model to assess the digital economy’s power on carbon emissions. This research has determined that the digital economy can significantly diminish carbon emissions, with green tech innovations and industrial transformation being key contributors. The spatial spillover effect was used for the digital economy to aid in lowering carbon emissions in adjacent districts and upgrading better environmental stewardship. The influence of the digital economy has better performance in lowering carbon emissions in mid-western China than in the eastern area. This paper deepens understanding of the drivers of low-carbon growth and the significance, mechanism and regional disparities of the digital economy’s effect on reducing carbon emissions. It offers valuable policy insights and guidance for globally achieving digital economy growth, reducing carbon emissions and reaching carbon peak and neutrality goals.

## 1. Introduction

Global warming is a significant challenge facing humanity. The hastened melting of glaciers worldwide, rising sea levels, prolonged droughts and increasing flooding are the primary indicators of frequent changes in the Earth’s climate [1]. These effects significantly hinder global sustainable development [2]. Global climate issues have escalated recently, intensifying the problem of global warming. As a result, many countries are trying to become low-carbon countries [3]. Being the world’s foremost emitter of carbon and the largest consumer of fossil fuels, China carries the dual obligation of minimising its carbon emissions and actively pursuing low-carbon development through domestic initiatives [4–6]. Resolving the apparent conflict between Chinese economic advancement and carbon emissions output is imperative while achieving the dual carbon goals in the Paris Agreement. This commitment applies to all parties involved and presents an invaluable opportunity for other nations, particularly developing countries [7]. And the widespread implementation of digital technology spearheads the global fourth industrial revolution. As a competitive emerging global economy, China continues to spur the development of its digital economy (DE). According to Zhang et al. [8], the China Academy of Information and Communication Technology reports that China’s DE experienced notable growth in 2022, reaching 50.2 trillion Chinese yuan (CNY), with a yearly increase of 10.32%. This upwards trajectory underscores the rising importance of the relationship between the DE and the national economy as it becomes increasingly integrated into all facets of society and the economy. The progression of the DE fosters the convergence of traditional and digital industries, propelling the transformation and modernisation of traditional sectors and fuelling the emergence of novel industries [9]. These new industries, which focus on low-carbon development, have substantially integrated with the real economy, promoting an inclusive green transformation and achieving better development [10]. The DE has become a crucial factor in high-quality economic development and addressing climate change. Therefore, it is important to explore the contribution of digital technology to energy conservation and emission reduction, as well as the reasons for regional differentiation. This can provide theoretical support for expanding the level of digital economy development and narrowing the development gap between regions. While we acknowledge the significant role of the digital economy in reducing carbon emissions, we must examine its specific impact mechanism and operating laws in this process. Additionally, we should consider any potential spatial spillover effects and regional heterogeneity between different regions. Analysing the above issues can help us understand the carbon emission reduction effect of the development of the digital economy. It can also provide a theoretical basis and empirical support for further promoting the digital economy to empower green and low-carbon development.

In summary, this paper makes three contributions: (1) Develop an assessment mechanism that considers three dimensions: infrastructure, application degree, and development degree of the digital economy. This will provide a more comprehensive portrayal of the digital economy’s development level in different provinces and regions. (2) Develop a theoretical model to validate the mechanism of carbon emission reduction through the digital economy. Determine the dual channels based on green technological innovation and upgrading of the industrial structure using the intermediary effect model. This will clarify the ways of reducing carbon emissions by developing the digital economy. (3) Analyse the impact of the digital economy on the process of carbon emission reduction using a spatial measurement model. This will provide the government with a basis for making decisions on promoting a low-carbon economy. The study will provide a theoretical basis for the government to develop a scientific strategy for the development of the digital economy and make informed decisions on carbon emissions reduction.

**Fig 1:**
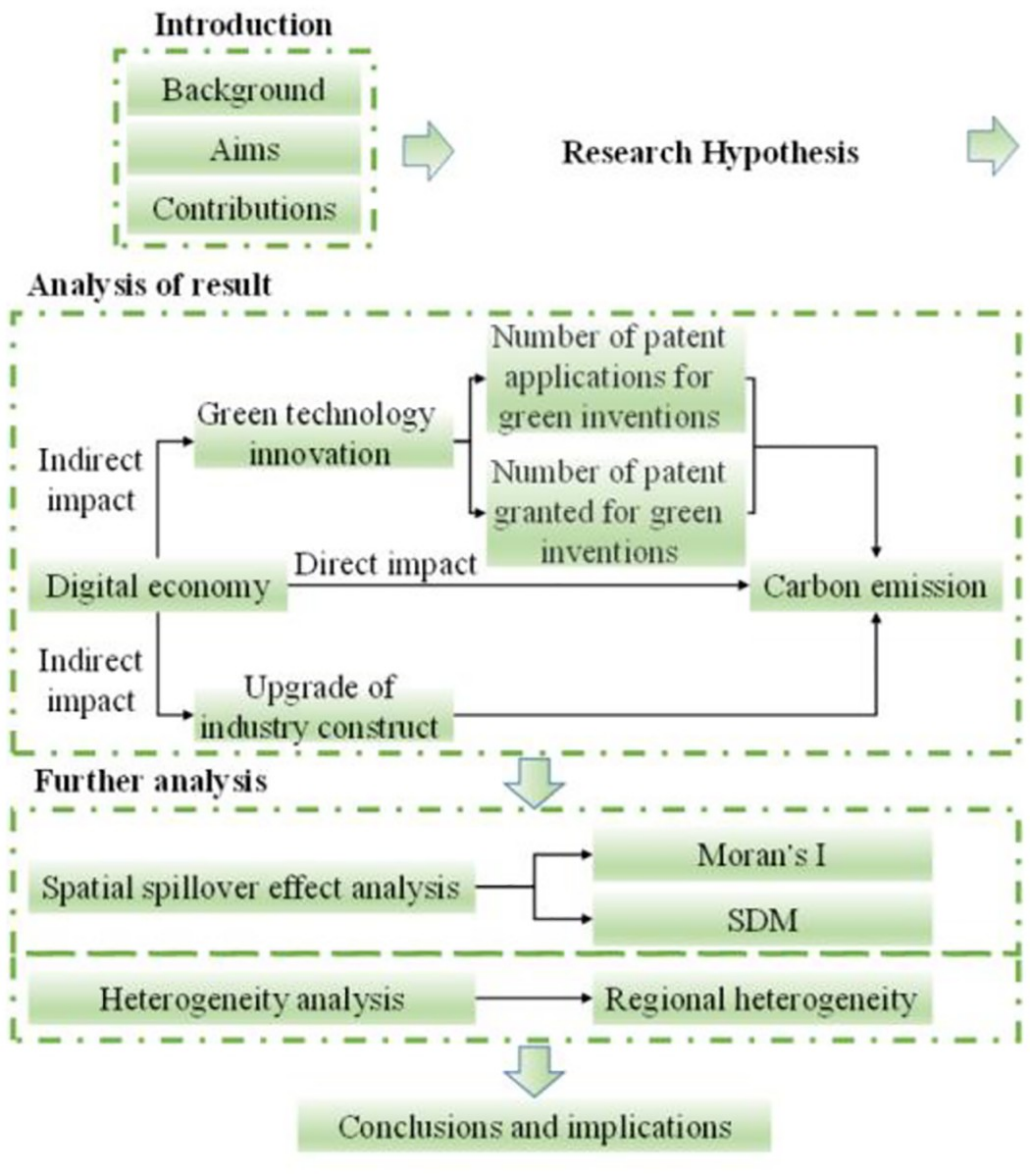
Technology road map.

This paper is structured into multiple sections. Section 2 presents the relevant mechanism and establishes the hypothesis for better analysis, while Section 3 introduces the collected data and used methods. Section 4 showcases the baseline regression results alongside mechanism testing and analysis, and Section 5 provides a supplementary analysis. The conclusions and implications are written in Section 6. Fig 1 presents the analytical framework used in this study.

## 2. Literature review

The importance of reducing carbon emissions has received increasing attention from scholars over the years. Previous studies have shown that carbon emissions are mainly influenced by economic growth [11, 12], technological advancement [13], urbanisation [14–16], industrial agglomeration [17, 18], energy demand [19], and carbon taxes [20]. In terms of the realisation pathway of carbon emission reduction, some scholars have argued that coal subsidy reforms have contributed to carbon emission reduction to a certain extent [21, 22]. On the other hand, Fischer and Newell [23] assessed the relative performance of carbon abatement policies using data from the US electricity sector. They conclude that emission pricing, emission performance standards and fossil energy taxation policies rank high in achieving emission reductions and that multi-policy combinations outperform single policies due to significantly lower costs. Qi and Cheng [24] argue that the establishment of a multi-level carbon emissions trading system is particularly important for carbon emission reduction, while Lin and Wang [25] argue that increases in urban employment and R&D intensity are also beneficial for carbon emission reduction. In addition, Shao et al. [26] extend the LMDI decomposition model to decompose and account for changes in energy-related industrial CO_2_ emissions (EICE). The authors emphasise that scaling up output increases EICEs, while industrial restructuring such as R&D inputs, R&D efficiency and investment intensity reduces EICEs. In addition, Dogan and Seker [27] investigated the relationship between clean energy and CO_2_ through causality tests. They observed a bidirectional causality between the two variables and found that clean energy use favours carbon emission reduction.

With the expanding scope of research related to emission reduction, some scholars have begun to study the impact of digital technology and digital economy on carbon emission reduction. These studies are mainly conducted in three dimensions: mechanism analysis, effect measurement and path selection. Firstly, Moyer and Hughes [28] initiated a study on the mechanism of the digital economy’s impact on carbon emissions. The authors found that DE generates carbon emissions by dynamically affecting economic and energy systems. Nonetheless, they concluded that DE had a limited impact on the decline of carbon emissions. Six years later, Zhou et al. [29] constructed a complete carbon analysis framework based on the input-output approach. They found that the DE industry is more environmentally friendly. Nevertheless, the DE industry still requires a large amount of electricity and carbon-intensive materials as intermediate production inputs, which generates a large amount of carbon emissions. Ran et al. [30] conclude that there is a moderating effect of the DE between the energy mix and carbon emissions. An increase in the level of DE can reduce the negative impact of energy structure on carbon emissions. Li et al. [31] further investigated that this moderating effect has significant regional heterogeneity in China, which is mainly manifested by the fact that this moderating effect is more significant in non-resource provinces in the eastern region, while it is not significant in resource provinces and the central and western regions.

## 3. Mechanism analysis and research hypothesis

### 3.1 Direct impact

The use of data has become a crucial factor in production. It is believed that the development of the DE can facilitate the efficient flow, scientific integration, and utilization of various factors, leading to a reduction in unnecessary resource and energy consumption [32]. This, in turn, can enhance the efficiency and productivity of social production while also reducing carbon emissions. The progress and penetration of technologies such as big data, cloud computing, artificial intelligence and mobile Internet are the foundation of the digital economy. Additionally, the digital economy can provide broader information dissemination channels and a more substantial knowledge dissemination effect. According to new growth theory, knowledge accumulation is a core factor in promoting technological innovation [33]. The digital economy’s knowledge diffusion effect will significantly enhance knowledge diffusion efficiency and improve the overall economy on a macro level. The application of more internet technologies to environmental protection and energy efficiency will promote research, development, and application of carbon emission reduction technologies, ultimately reducing carbon emissions. Accordingly, the assumption here is:

Hypothesis H1: The DE can effectively reduce carbon emissions

### 3.2 Indirect impact

Antweiler et al. [34] have identified three effects of environmental pollution which include technology, structure, and scale. This study examines the potential of the digital economy to reduce carbon emissions by investigating its impact on the innovation of green technology and the upgrading of industrial structure. Firstly, from the technology effect, the DE essentially hinges on the integration of advanced technologies, particularly artificial intelligence and big data. These information technologies form key components of low-carbon and sustainable technologies [35]. Green technology constitutes a new modern technology system that complements ecological and environmental systems by transforming traditional industrial sectors and encouraging enterprises to regularly update their production technologies and equipment [36]. The implementation of cleaner, more efficient, and greener practices throughout all production stages has the potential to achieve a green boost for the entire industrial chain. This, as a result, has the potential to promote the shift of industries from highly polluting, low-value-added sectors to high-value-added and eco-friendly sectors and decrease the share of non-green industries in the total output of the national economy. Du and Li [37] have reported that energy conservation and emission reduction through this technology can assist in optimal resource allocation and minimal emissions, thus making a contribution to the improvement of the environmental situation. Second, in terms of structural impact, the upgrading of an industrial structure involves systematically moving from lower to higher levels of industrial structure, and is typically accompanied by a continuous rise in the share of tertiary industry. With the accelerated advancement of information technology, a productive digital economy is being established, characterized by openness, collaboration and sharing, such as the Internet and the Internet of Things. This can be employed to prompt industrial upgrading by promoting resource-sharing between sectors and enterprises using the dimension of enterprise scalability and competitiveness, and integrating it into the production process, whilst harmonizing it with previously existing production factors. The pursuit of high-end industrial development requires a multi-faceted approach, featuring the exploration at connotations, discovery on spaces, and the creation on other domains. During China’s industrial development, the production factors of Chinese industrial enterprises have shifted from low-end, to high marginal profit industries, as industrial development trends increasingly towards those oriented towards green and environmental protection [38].

On this basis, an attempt is made to introduce all these factors into the research framework so as to develop a theoretical model of the multi-pathway influence on carbon emissions. A production function, including energy consumption, is first constructed as shown in Eq (1):

$$Y_{i}=A_{i}L_{i}^{\alpha }K_{i}^{\beta }E_{i}^{1-\alpha -\beta }$$ (1)

$$C_{i}=\frac{E_{i}^{1-\alpha -\beta }}{Y_{i}}=\frac{E_{i}^{\theta }}{Y_{i}}=\frac{1}{A_{i}L_{i}^{\alpha }K_{i}^{\beta }},\theta =1-\alpha -\beta$$ (2)

where *Y*_*i*_, *C*_*i*_, *A*_*i*_, *L*_*i*_, *K*_*i*_ and *E*_*i*_ represent industry i’s actual output, carbon emissions, green technology innovation, labour input, capital input and energy consumption.

The innovation of green technology *A*_*i*_ is a function that experiences growth as the DE, *μ*, advances:

$$A_{i}=f\left(\mu \right)$$ (3)

Bringing Eq (3) into Eq (2):

$$C_{i}=\frac{E_{i}^{\theta }}{Y_{i}}=\frac{1}{f\left(\mu \right)L_{i}^{\alpha }K_{i}^{\beta }}$$ (4)

From Eq (4), it follows that as the DE, *μ*, increases, green technology innovation, *A*_*i*_, increases while sectoral carbon emissions decrease.

This study next extends the model to a two-sector version, with the lower sector, *C*_*1*,_ called the low-carbon emitting sector and the higher sector, *C*_*2*_, called the high-carbon emitting sector.

According to some scholars, low-carbon industries generally refer to any industry characterised by low carbon emissions or committed to reducing carbon emissions. Some scholars refer to high-energy-consuming and high-polluting industries as high-carbon industries due to their high carbon emission intensity and potential for emission reduction. According to the Statistical report on National Economic and Social Development in 2010 in China, the six energy-intensive industries are: chemical raw materials and chemical manufacturing; non-metal mineral product manufacturing; smelting and rolling processing of ferrous metals; petroleum processing; coaling and nuclear fuel processing; and production and supply of electric and thermal power (National Bureau of Statistics of the People’s Republic of China (2011)). These six energy-intensive industries use most of the energy consumed by the industrial sectors. As shown in Eq (5):

$$C_{2}/C_{1}=\varepsilon >1$$ (5)

The evolution of the industrial structure of a region is influenced by the DE (*μ*) in that region:

$$upg=Y_{1}/Y_{2}=g\left(\mu \right)$$ (6)

$$C=\frac{E}{Y}=\frac{E_{1}^{\theta }+E_{2}^{\theta }}{Y_{1}+Y_{2}}=\frac{Y_{1}C_{1}+Y_{2}C_{2}}{Y_{1}+Y_{2}}$$ (7)

Substituting Eqs (5) and (6) into Eq (7):

$$C=\frac{Y_{1}C_{1}+Y_{2}C_{2}}{Y_{1}+Y_{2}}=\frac{\left(Y_{1}/Y_{2}\right)C_{1}+C_{2}}{Y_{1}/Y_{2}+1}=\frac{upg+\varepsilon }{upg+1}\times C_{1}=\frac{g\left(\mu \right)+\varepsilon }{g\left(\mu \right)+1}\frac{1}{f\left(\mu \right)L_{1}^{\alpha }K_{1}^{\beta }}$$ (8)

From Eq (8), since *ε*>1, as the DE (*μ*) increases, the larger the *upg*, the smaller the *C*. When the low carbon emission sectors increase in proportion, the total district’s carbon emissions will decrease. This paper proposes the following hypotheses based on this mechanism.

Hypothesis H2: The green technology innovation path can be improved by utilising the DE, leading to a carbon emission reduction.
Hypothesis H3: Industrial structure can be upgraded by utilising the DE, thereby reducing carbon emissions.

### 3.3 Spatial spillover effects

The DE, facilitated by network connectivity, plays a significant part in exchanging and disseminating information, presenting novel prospects for restructuring economic production’s spatial distribution [39]. Although the exchange of traditional economic information is often limited by various constraints, such as lengthy geographical distances, sluggish transmission channels and high time costs, the DE operates through information networks and is not bound by conventional limitations. It facilitates the swift exchange of knowledge, information and economic activities at a lower cost, thereby promoting the uninhibited flow of production factors over vast distances. The DE strengthens the expansion of capital and technological extensibility and has emerged as a crucial factor for determining the spatial layout of economic activities [40]. The geographical concentration of carbon emissions in different regions underscores the importance of examining the DE. Contemporary information networks, serving as the main conduit, are vital to their functionality, depending substantially on digital expertise and data as essential production components. The DE is notable for its capacity to amalgamate and orchestrate diverse elements, allowing it to transcend geographical constraints, facilitating specialisation and collaboration between regions, generating spatial consequences and making it apparent that its influence in one area has a discernible effect on carbon emissions in other regions [41]. The following hypothesis is proposed:

Hypothesis 4: The carbon reduction effect of the DE exhibits a spatial spillover.

## 4. Methodology and data

### 4.1 Methodology

Under hypothesis H1, this thesis obtains a bidirectional fixed-effect baseline model (9):

$$\begin{array}{c} \text{ln}Y_{it}=\beta_{0}+\beta_{1}de_{it}+\beta_{2}fin_{it}+\beta_{3}fdi_{it}+\beta_{4}pop_{it}+\beta_{5}urb_{it}+\beta_{6}inf_{it}\\ +\beta_{7}gdp_{it}+\beta_{8}sc_{it}+\beta_{9}er_{it}+\beta_{10}lnY_{it-1}+\delta_{i}+\gamma_{t}+\varepsilon_{it} \end{array}$$ (9)

Eq (1) includes explained variables that represent the region’s carbon emission level in the year, as measured by total carbon emission (CE) and per capita carbon emission (PCE). The primary explanatory variable, *de*_*it*_, reflects the DE in region *i* during year *t*. Additional vectors include a set of control variables that have the potential to impact CEs and CE levels lagged by one period (lnY_it-1_). Moreover, *δ*_*i*_ indicates area-fixed effects, *γ*_*t*_ represents time fixed effects and *ε*_*it*_ serves as the error term.

The examination relied on hypotheses H2 and H3, incorporating mediating effects. Given the endogeneity difficulties associated with the third step of the conventional intermediary effect test, this study draws on previous research by Jiang [42] and solely utilises the second step of the three-step process to evaluate its mechanism. The examination of how green technology innovation and upgrades in industrial structure affect CEs relies mainly on existing literature and logical inferences, and the econometric model (10) is constructed as outlined below:

$$\text{ln}\;Z_{it}=\beta_{0}+\beta_{1}de_{it}+\beta_{2}X_{it}+\delta_{i}+\gamma_{t}+\varepsilon_{it}$$ (10)

ln *Z*_*it*_ represents the innovation of green technology and industrial structure optimisation, while *X*_*it*_ comprises control variables.

The DE fosters cooperation and integration of regional resources, drives technological progress and supports rational allocation of production resources, leading to noticeable advancements in regional energy efficiency [43]. Multiple linear regressions based on general panel data may suffer substantial deviation. A viable solution would involve integrating Lesage’s Durbin model (2009) with the standard regression Eq (1) and conducting empirical research to develop a spatial Dubin model with dual fixed effects. The model can be expressed as Eq (11):

$$\text{ln}\;Y_{it}=\beta_{1}W_{ij}\text{ln}Y_{it}+\beta_{2}de_{it}+\beta_{3}W_{ij}de_{it}+\beta_{4}X_{it}+\beta_{5}W_{ij}X_{it}+\delta_{i}+\gamma_{t}+\varepsilon_{it}$$ (11)

*W*_*ij*_ is a spatial weight matrix of 30 × 30, and two spatial matrices are employed here for experimentation. How the DE touches on CEs is not confined to adjacent bordering regions, and mutual influence may exist even when not adjacent. Therefore, under the 0–1 spatial adjacency weight matrix, 1 means that two provinces are adjacent, and 0 is not adjacent [44]. This study constructs a spatial economic geographic distance weight matrix following the approach suggested by Hu et al. [45], combining the spatial weight of geographic and economic distances. The spatial economic geographic distance weight matrix is derived using the following Eq (12):

$$W_{ij}^{d}=\frac{1}{d_{ij}},W_{ij}^{e}=\frac{1}{{\bar{GDP}}_{i}-\bar{GDP_{j}}},W_{ij}^{d-e}=\frac{{\bar{GDP}}_{i}-\bar{GDP_{j}}}{d_{ij}}$$ (12)

where *d*_*ij*_ is the distance between cities. ${\bar{GDP}}_{i}$ and ${\bar{GDP}}_{j}$ are the average 2011–2020 deflating GDP of provinces *i* and *j* to obtain the economic geographic distance weight matrix $W_{ij}^{d-e}$. *X*_*it*_ is a series of control variables.

### 4.2 Data

Explained variables: This article selects each province in China’s total and per capita CEs as the metrics. Wu and Guo [46] proposed that total CE can be computed by analysing 17 types of energy usage contributing to each province’s carbon dioxide emissions. The total carbon dioxide can be calculated after discounting and adding the carbon emission factors from different energy sources. The PCE is based on the proportion of total CEs to the total population. The formula for calculating CO_2_ emissions is shown in Eq (13):

$$CE=\sum_{i}^{17}A_{i}\times N_{i}\times CC_{i}\times O_{i}\times B$$ (13)

where *CE* represents CO_2_ emissions and *i* represents fossil energy type. *A*_*i*_ represents energy consumption, and *N*_*i*_ represents low-level heat generation. The CE factor (*CC*_*i*_) is based on the guidelines provided by IPCC in 2006. The carbon oxidation factor (*O*_*i*_) is considered, along with the mass ratio of CO_2_ molecules to elemental carbon (*B*), which equals 44/12.

Explanatory variables: Although the DE is developing, its quantitative indicators and credibility are lacking. Accurately measuring this sector’s comprehensive development at China’s municipal and provincial levels is challenging. As infrastructure, application degree and level of development are all vital components of digitisation, the evaluation mechanism proposed in this study draws on Zhao et al. [47], which utilises principal component analysis to establish a framework for analysing the state of DE. This novel approach provides scientific evidence of its potential to facilitate the advancement and transformation of economic and societal structures. Table 1 illustrates the framework for analysing the state of DE.

**Table 1:** Evaluation index system for measuring the DE.

Indicators	Primary Indicators	Secondary Indicators	Unit
Digital Economy Development Level	Digital Economy Infrastructure	Internet broadband access ports	10,000
		Mobile Phone Penetration Rate	Department/100 people
		Fibre-optic cable line length	Kilometres
		Cell phone exchange capacity	million households
	The degree of DE application	Number of websites	million
		Total number of telecom services	billion CNY
		China Digital Financial Inclusion Index	/
	Degree of DE Development	R&D investment intensity	million
		Software Product Revenue	million CNY
		Information employed persons	million

**Fig 2:**
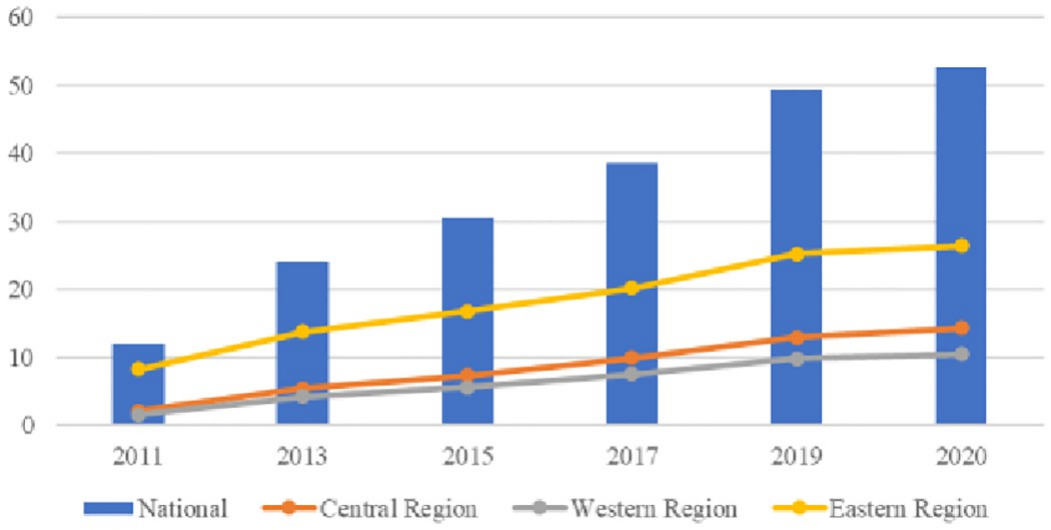
Trends in the level of development of China’s digital economy from 2011 to 2020.

The level of DE in 30 provinces across China from 2011 to 2020 is assessed based on the DE indicators calculated in this section. To comprehensively analyse regional differences in China’s DE, this study uses the division criteria provided by the National Bureau of Statistics (NBS) to divide the measurement sample into three major regions. A trend graph was drawn to facilitate a comparative assessment of the sample measurement averages for each region during the study period, as shown in Fig 2. The overall trend shows that DE in China rose from 2011 to 2020. Regarding specific regions, the growth of DE is relatively high in the eastern region, followed by the central region; DE is relatively slower in the western region.

Mechanism variables: When exploring the innovation of green technology, an evaluation of the number of green patent applications (GPAP) and granted green patents (GPAU) is crucial [48]. The current study collected data on GPAP and GPAU in each region by utilising the green application information disclosed by the State Intellectual Property Office and IPC codes and authorisation/application dates. This method enabled a comprehensive analysis of the volume of GPAP and granted patents in each area. The upgrading of industrial structures (UPG) can be expressed as the scale of the tertiary sector to the secondary sector in the economy.

Control variables: This research introduces additional control factors and inspects the role these variables played in CEs to prevent any discrepancies in the model estimation outcomes caused by the inadequate number of variables. (1) The financial development level (FIN) is determined by the level of the balance of various loans that were provided by banking financial institutions to the GDP in the areas [49]. (2) Foreign direct investment (FDI) is calculated based on the performance of overseas direct investment. (3) Population size (POP) is expressed as the number of individuals in a region at the end of the year. (4) Urbanisation (URB) is the ratio between the urban and total populations. (5) Infrastructure (INF) is the number of road miles/provincial area. (6) Economic development level (GDP) refers to Henderson et al. [50], using night-time lighting data as one of the control variables. (7) Social consumption (SC) is the total social retail sales. (8) Environmental regulation (ER) is determined by calculating the ratio between the investment made in controlling industrial pollution and the value included by the secondary industry.

### 4.3 Sample and data sources

The research centred on China’s 30 provincial administrative districts from 2011 to 2020. This thesis manually compiled data on green technology innovation from the China Digital Inclusive Finance Index sourced from the Digital Finance Research Centre of Peking University. Economic development data was from VIIRS/DNB data obtained from the official website of the US National Institute of Atmospheric and Oceanic Administration. This thesis collected the remainder of provincial data from the China Statistical Yearbook and statistical yearbooks published by each province. In the event of missing data, this thesis applied interpolation methods to address the gaps. Table 2 shows the descriptive statistics of each variable.

**Table 2:** Descriptive statistics.

Type	Indicators	Symbol	N	Mean	Sd	Min	Max
**Explained variable**	Total Carbon Emissions	*CE*	300	8.303	0.661	6.506	9.564
	CEs per Capita	*PCE*	300	1.201	0.557	0.575	3.465
**Explanatory variables**	Digital Economy	*DE*	300	1.100	0.732	0.077	4.055
**Mechanism variables**	Number of Green Patent Applications	*GPAP*	300	7.943	1.308	3.434	10.800
	Number of Green Patents Granted	*GPAU*	300	7.559	1.361	3.219	10.870
	Industrial Structure Upgrade	*UPG*	300	1.219	0.696	0.518	5.297
**Control variables**	Financial Development	*FIN*	300	1.446	0.942	0.655	15.830
	Foreign Direct Investment	*FDI*	300	0.393	0.379	0.0378	1.842
	Population Size	*POP*	300	8.206	0.736	6.342	9.443
	Urbanisation	*URB*	300	59.010	12.220	35.030	89.600
	Infrastructure	*INF*	300	38.980	31.290	1.342	128.800
	Economic Development	*GDP*	300	13.680	0.692	11.640	15.080
	Social Consumption	*SC*	300	0.932	0.453	0.019	2.461
	Environmental Regulation	*ER*	300	0.003	0.003	4.44e-05	0.025

## 5. Results

### 5.1 Baseline regression results

This study used a two-way fixed effects regression model to evaluate how the DE affects CEs while considering constant individual differences and the macroeconomic environment. The outcomes in Table 3 indicate a substantial adverse effect on CE and PCE due to the DE, with a significance level of 1%. Without the control variables, CE and PCE decrease by 0.131% and 0.158% for every 1% increase in DE. With the control variables, for every 1% increase in DE, CE and PCE decrease by 0.104% and 0.074%, which confirms hypothesis H1 and aligns with the opinions of other researchers [31]. They also believe that the DE can significantly reduce CEs. The DE has accelerated the digitisation and informatisation of society and improved the management and utilisation of enterprise resources. It promotes low-carbon, eco-friendly and clean energy consumption models and fosters green and low-carbon industrial development. With this progress, industries can transform and adjust, significantly reducing CEs and successfully moving towards the ‘30–60’ double carbon goal.

**Table 3:** Benchmark regression.

Variables	lnCE	lnCE	lnPCE	lnPCE
lnDE	-0.131***	-0.104***	-0.158***	-0.074***
	(0.023)	(0.030)	(0.019)	(0.016)
lnFIN		-0.006***		-0.004***
		(0.002)		(0.001)
lnFDI		0.041***		0.028***
		(0.009)		(0.007)
lnPOP		0.000***		-0.328**
		(0.000)		(0.123)
lnURB		0.005***		-0.002
		(0.002)		(0.002)
lnINF		0.003***		0.001***
		(0.001)		(0.000)
lnGDP		0.071**		0.000*
		(0.033)		(0.000)
lnSC		0.042**		0.014
		(0.018)		(0.010)
lnER		-4.525		-0.961
		(3.240)		(1.980)
L.lnCE		0.000***		
		(0.000)		
L.lnPCE				0.444***
				(0.033)
cons	8.336***	0.000	0.170***	2.300**
	(0.009)	(0.000)	(0.008)	(1.005)
Time FE	Yes	Yes	Yes	Yes
Area FE	Yes	Yes	Yes	Yes
N	300	300	300	300
R^2^	0.1114	0.5115	0.1995	0.6453

Note:

*** p<0.01,

** p<0.05,

* p<0.1;

standard errors in parentheses, same as the table below.

### 5.2 Endogeneity test

While this paper strives to extenuate various factors affecting DE and CEs, certain unobservable factors still exist, leading to endogeneity.

The instrumental variables method is utilised for testing to prevent any impact stemming from endogeneity. Firstly, the effectiveness of the DE functionality is inherently connected to its application’s extensiveness. Imperfections in digital hardware and software, along with delayed adoption and limited development of digital technology, may cause a time lag in its reducing CO_2_ emissions effect. A systematic GMM two-step regression analysis was conducted with a one-period lag in the DE as an instrumental variable. The research results are presented in columns (1) and (2) in Table 4. Evident in Table 4, AR(1) is less than 0.05, AR(2) is greater than 0.1, and the Sargan test is greater than 0.1, all of which do not reject the null hypothesis, indicating that the instrumental variables in the model are valid. This research incorporates the findings of Huang et al. [51]. Secondly, the paper adopts the treatment provided by Nunn and Qian [52], creating a 2SLS regression analysis for the interaction term between the fixed telephone count per 100 individuals in each province in 2002 and the previous year’s national Internet investment amount (over time). The findings are shown in columns (3) and (4) of Table 4. The aforementioned statistical evidence reveals that DE progression can significantly reduce CEs. The similarity of these conclusions to the overall outcomes of the data reinforces the dependability of the typical regression results.

**Table 4:** Endogenous test.

Variables	CE	PCE	CE	PCE	DE	DE
DE	-0.491*** (0.186)	-0.698*** (0.211)	-0.970*** (0.331)	-0.819*** (0.167)		
CE					-0.101 (0.064)	
PCE						-0.061 (0.060)
cons	-6.156***(0.927)	-20.562*** (5.609)	-7.469*** (2.494)	3.012*** (0.902)	2.079** (0.798)	3.855 (2.905)
AR(1)	0.029	0.034				
AR(2)	0.957	0.184				
Sargan test	0.111	0.621				
Control variables	Yes	Yes	Yes	Yes	Yes	Yes
N	270	270	300	300	300	300

As per the prevailing expectation, DE’s growth and state are inextricably linked to the state of CEs, and the government’s ‘30–60’ dual carbon target has motivated businesses to improve their digital capabilities. The current study utilised the impact of the DE regression analysis to eliminate the possibility of any potential reverse causality bias. The outcomes have brought to light a remarkable association between its development and various economic features of diverse provinces in China. Such outcomes suggest that this influence cannot be regarded as entirely exogenous. No noteworthy correlation was discovered between the DE and either CE or PCE. By examining the impact of DE on the environment, the study’s estimated findings have partially negated the prospect of reverse causation here.

### 5.3 Robustness tests

Firstly, the previous regression analysis employed CE and PCE as the variables under investigation. For the purpose of robustness testing, this paper also measures carbon emissions intensity (CEI). CEI is defined as the total carbon emissions expressed as a proportion of GDP. This approach is consistent with the measure proposed by Dong et al. [53] on the CEI. The analysis in column (1) of Table 5 indicates that upon replacement of the explained variables, the model still exhibits significant negative coefficients at a 1% threshold, thereby preserving the consistency with the underlying regression analysis. This kind of economy can mitigate CEs considerably despite variations in the explained factors.

**Table 5:** Robustness test.

Variables	CEI	CE	PCE
DE	-0.375*** (0.027)		
SDE		-0.025* (0.013)	-0.025*** (0.009)
cons	0.756*** (0.265)	-0.036 (1.314)	-1.564*** (0.291)
Control variables	Yes	Yes	Yes
Time FE	Yes	Yes	Yes
Area FE	Yes	Yes	Yes
N	300	300	300
R^2^	0.8678	0.5349	0.6465

Secondly, this paper discusses the replacement of the method used to measure the development level of the DE. The approach taken is based on the work of Chen and Wang [54] and the Statistical Classification of the Digital Economy and its Core Industries (2021) (NBS Decree No.33). Based on the principle of homogeneity of the National Economic Industry Classification (GB/T47542017), this reclassifies industry categories in the National Economic Industry Classification that are consistent with the characteristics of the digital economy industry and aimed at providing digital products (goods or services). The core industries of the digital economy are determined by identifying the major (two-digit) industries that account for more than 50% of the subcategories. The four major industries selected are computer, communication and other electronic equipment manufacturing, telecommunication, radio and television broadcasting and satellite transmission services, Internet and related services, and software and information technology services. These industries are then matched with the industries in the WIOT. Finally, the integration of these factors using the entropy weight method results in a new measure of the level of development of the DE (SDE). The findings demonstrate a continued negative correlation between the DE and CEs after substituting the explanatory variables, thus aligning with the baseline regression outcomes; hence, it can be inferred that the advancement of DE continues to reduce CEs despite the explanatory variable replacement.

### 5.4 Mechanism test

The objective is to probe into the mechanism of reducing carbon dioxide emissions in DE by scrutinising the significance of green technology innovation and industrial optimisation. This study draws upon the aforementioned theoretical analysis to investigate how these factors influence CEs. The outcomes of the regression analysis are presented in Table 6. Prior research predominantly assessed that the green technology innovation process will be smoother with more GPAP. This study undertakes a comprehensive assessment of the mechanism behind green technological innovation to avoid relying solely on a single indicator. For this purpose, two indicators, namely green invention patent application amounts (GPAP) and green invention patent grant quantities (GPAU), are leveraged to analyse green technology innovation role mechanisms. This study applied a one-period lag to DE to determine the mechanism of granting green patents due to the long delay in the granting process. The results indicate that DE positively affects both GPAP and GPAU. For every 1% increase in DE, GPAP and GPAU increase by 0.106% and 0.857%, respectively. This finding is similar to [40], who argued that DE would significantly increase green technology invention. It is worth noting that DE has a more pronounced impact on the latter. Advancing green technology is of immense importance in curbing CEs in China. Technological breakthroughs can enable the development of eco-friendly productivity, promote the adoption of low-carbon lifestyles and production approaches and foster clean energy production and energy structure reforms. This situation will lead to sustainable reductions in energy consumption and CO_2_ emissions. The green technology innovation can unlock the full potential of the ‘technology dividend’ effect through industry collaborations, the amplification of technology chain effects and bolstering regional cooperation, ultimately reducing the greenhouse impact of CO_2_ and other gases, thereby affirming hypothesis H2 and previous research [55, 56]. The third column of data confirms a noteworthy increase in industrial structure upgrading. For every 1% increase in the development of the DE, the UPG increases by 0.159%. The DE changes production modes, leading to enhanced cooperation among diverse stakeholders. With a more extensive division of labour within the industrial chain, the DE accelerates the shift of conventional industries to more sustainable and environmentally friendly production modes. As a result, it reduces carbon dioxide emissions while driving their development in a cleaner and greener direction. Based on these findings, hypothesis 3 was successfully confirmed [57].

**Table 6:** Intermediary mechanism test of DE on environmental pollution.

Variables	(1)	(2)	(3)
	GPAP	GPAU	UPG
DE	0.106*** (0.030)		0.159*** (0.053)
l.DE		0.857*** (0.177)	
cons	-4.391*** (0.899)	-13.888*** (1.683)	14.129*** (0.762)
Control variables	Yes	Yes	Yes
Time FE	Yes	Yes	Yes
Area FE	Yes	Yes	Yes
N	300	270	300
R^2^	0.8943	0.8715	0.8265

## 6. Further research

### 6.1 Spatial spillover effects

This subsection investigates the reciprocal impact of the spatial correlation of DE and CE under the econometric model was analysed [58, 59]. The analysis commences with the computation of spatial autocorrelation coefficients for each year under the spatial adjacency matrix through Moran’s I index method, as exhibited in Table 7. Findings indicate a significant spatial autocorrelation in CE intensity and DE growth indices across all provinces and regions in China during the 2011–2020 period. The Moran’s I index was greater than 0, indicating that these variables tend to cluster in spatial distribution. Though the spatial clustering state seems stable overall, volatility appears in values.

**Table 7:** Moran index.

Year	DE			CE			PCE		
	Moran’s I	Z	P	Moran’s I	Z	P	Moran’s I	Z	P
2011	0.174	1.810	0.035**	0.272	2.814	0.002***	0.404	4.033	0.000***
2012	0.170	1.776	0.038**	0.229	2.430	0.008***	0.370	3.641	0.000***
2013	0.136	1.461	0.072*	0.226	2.430	0.008***	0.418	4.101	0.000***
2014	0.111	1.261	0.104	0.237	2.514	0.006***	0.393	3.843	0.000***
2015	0.115	1.286	0.099*	0.216	2.299	0.011**	0.354	3.547	0.000***
2016	0.115	1.287	0.099*	0.205	2.206	0.014**	0.321	3.248	0.001***
2017	0.121	1.333	0.091*	0.165	1.822	0.034**	0.245	2.638	0.004***
2018	0.120	1.322	0.093*	0.171	1.925	0.027**	0.259	2.803	0.003***
2019	0.154	1.605	0.054*	0.174	1.919	0.028**	0.266	2.886	0.002***
2020	0.147	1.551	0.060	0.197	2.119	0.017	0.294	3.197	0.001

This study employs a spatial econometric model to simulate the relationship and spatial effects of digital economic development on carbon emissions, based on the test results of spatial autocorrelation. The text describes the process of selecting a spatial and temporal double-fixed effects SDM model. Firstly, the LM test is used to determine the specific type of model. Secondly, the Hausman test is used to decide whether to construct a fixed effects model. Thirdly, the LR test was employed to ascertain whether the SDM model reduces to the SAR and SEM models and to select suitable fixed effects. Finally, the text concludes that a spatial and temporal double-fixed effects SDM model is the best choice. The test results are presented in Table 8.

**Table 8:** Spatial correlation test of residuals based on OLS estimation results.

Spatial matrix type Variables	W1		W2	
Type of test	CE	PCE	CE	PCE
LM-lag	83.337***	45.906***	27.085***	7.958***
Robust LM-lag	9.404***	2.815*	12.730***	3.175*
LM-error	250.627***	198.797***	22.600***	30.633***
Robust LM-error	176.694***	155.706***	8.244***	25.850***
Hausman test	48.33***	519.33***	36.83***	36.62***
LR-SDM-SEM	60.43***	62.49***	30.20***	22.21***
LR-SDM-SAR	53.72***	58.79***	32.04***	25.68***
LR-both-ind	59.45***	60.46***	46.17***	31.82***
LR-both -time	737.71***	687.95***	658.87***	655.32***

Table 9 presents the outcomes of the spatial econometric regressions using two distinct spatial weights. Firstly, sigma2_e significantly indicates that the spatial Durbin model fits the data well. The findings indicate a notable adverse correlation was established utilising both spatial weight matrices, attaining a 1% significance level, between DE and two types of CEs. Under the spatial proximity weighting matrix, for every 1% increase in the DE, CE and PCE decrease by 0.240% and 0.178%; under the spatial economic geographic distance weighting matrix, for every 1% increase in the DE, CE and PCE decrease by 0.233% and 0.184. Consequently, fostering the DE can significantly curtail provincial CEs in China. Scrutiny of the spatial interaction term’s regression coefficients of the two unveils a negative W.de. coefficient. This finding indicates that besides endogenous interaction effects of CEs between provinces, spatial spillover effects can also come from local DE efforts, helping to diminish CEs in neighbouring regions.

**Table 9:** Estimation results of spatial dobbin model for bidirectional fixed effects.

Spatial matrix type Variables	W_1_		W_2_	
	lnCE	lnPCE	lnCE	lnPCE
DE	-0.240***	-0.178***	-0.233***	-0.184***
	(0.040)	(0.031)	(0.034)	(0.044)
W.DE	-0.979***	-0.785***	-0.345***	-0.242**
	(0.347)	(0.284)	(0.079)	(0.107)
rho	-0.883***	-0.973***	-0.242**	-0.287***
	(0.268)	(0.268)	(0.100)	(0.101)
sigma2_e	0.005***	0.005***	0.005***	0.005***
	(0.000)	(0.000)	(0.000)	(0.000)
Control variables	Yes	Yes	Yes	Yes
Time FE	Yes	Yes	Yes	Yes
Area FE	Yes	Yes	Yes	Yes
R^2^	0.447	0.1078	0.6542	0.1666

Table 10 shows that the complete effect demonstrates an adverse correlation of 1% under both spatial weight matrices, emphasising the DE’s efficacy in diminishing CEs. The two matrices’ direct effect is also extremely negative. The DE in the region culminates in an enhanced curbing impact on emissions, hence attesting to the fundamental regression inference. This is not entirely consistent with some of the findings of Li and Wang [39], who confirm that the spatial spillover effect of the DE on CEs is inverted U-shaped. This result may be because they distinguish between the short and long term. The indirect effect is negative under the two spatial weight matrices, indicating a decreasing influence on local CEs. It also has a noticeable spillover effect across varied regions, enhancing ecological environment quality in other regions. The outcome corroborates the hypothesis H4 mentioned previously.

**Table 10:** SDM.

Spatial matrix type	W_1_		W_2_	
Explained variables	lnCE	lnPCE	lnCE	lnPCE
Direct effect	-0.213***	-0.155***	-0.219***	-0.172***
	(0.040)	(0.032)	(0.035)	(0.044)
Indirect effects	-0.435**	-0.333**	-0.243***	-0.154***
	(0.203)	(0.161)	(0.068)	(0.086)
Total effect	-0.648***	-0.488***	-0.462***	-0.326***
	(0.213)	(0.162)	(0.079)	(0.104)
Control variables	Yes	Yes	Yes	Yes
Time FE	Yes	Yes	Yes	Yes
Area FE	Yes	Yes	Yes	Yes
N	300	300	300	300

### 6.2 Regional heterogeneity

The development performance of the eastern region differs significantly from that of the midwestern region regarding the DE, industrial structure and green technology invention [39]. This study partitions the country into two distinct zones, namely the eastern region and midwestern region, to investigate the regional heterogeneities in how DE optimises environmental development. Table 11 shows that the DE remarkably impedes CEs, indicating that the effect of the midwestern region is more significant than the eastern region. This discrepancy may be due to the lower economic development in the midwestern region, which results in higher reliance on traditional resources and a lower energy utilisation rate. The rise of digital technology has enabled market players to comprehend market dynamics and price trends better, leading to efficient resource allocation. Adopting digital technology in industrial production can aid in reducing energy usage, improving energy utilisation and decreasing CEs. Empirical observations reveal that China’s DE exerts a more substantial impact on restraining CEs in the midwestern region, thereby producing more marginal utility and providing a stronger latecomer advantage in the pursuit of the ‘30–60’ dual carbon goal [60].

**Table 11:** Heterogeneity analysis based on spatial characteristics.

Variables	Eastern Region		Midwest Region	
	CE	PCE	CE	PCE
DE	-0.090*** (0.026)	-0.050*** (0.015)	-0.194*** (0.051)	-0.084*** (0.022)
cons	8.711*** (0.916)	4.479*** (1.158)	7.627*** (0.908)	-1.565*** (0.623)
Control variables	Yes	Yes	Yes	Yes
Time FE	Yes	Yes	Yes	Yes
Area FE	Yes	Yes	Yes	Yes
N	120	120	180	180
R^2^	0.2943	0.5030	0.7322	0.2720

## 7. Discussion, conclusion and policy implications

### 7.1 Discussion

This research offers five notable contributions to existing literature. (1) This study presents an analytical model to examine the nexus between DE and CEs at a theoretical level. Based on the concepts of digital progress and DE proposed by Li and Wang [39]. This study incorporates energy consumption, green technology invention and industrial structure enhancement into the production function model. By extending their model into a dual-sector format, this study infer that the DE can affect CEs directly and indirectly. (2) This study suggests that DE may have a negative impact on CE and PCE, which is consistent with some existing studies [61, 62].These studies similarly concluded that the growth of the digital economy would reduce carbon emissions. (3) Additionally, this study indicates that DE can negatively affect CE and PCE by promoting green technology innovation and upgrading industrial structure. This finding aligns with previous studies that have shown DE to be effective in achieving emission reduction targets through technological innovation and industrial upgrading [63]. (4) This study concludes that DE can reduce CE not only in specific regions but also in local and neighbouring areas. This finding is consistent with previous studies, indicating that DE has spatial spillover effects [64]. It also has a noticeable spillover effect across varied regions, enhancing ecological environment quality in other regions. (5) This study reveals that the central and western regions have a greater advantage in reducing carbon emissions, which is referred to as the ’latecomer’s advantage’ at the regional level. These findings offer a new perspective and reference for the formulation of regional policies aimed at reducing carbon emissions.

### 7.2 Conclusion and policy implications

The interconnectedness between the DE and the real economy has arisen as a new catalyst and driver for creating a sturdy power for long-term and sound economic growth in China. Despite ‘carbon peak’ and ‘carbon neutral’ programmes, the DE in mitigating low-CEs is significant. The two-sector model is established to reveal the new mechanism of DE for CE reduction, and empirical tests are conducted. The DE’s effect on CEs and its mechanism are empirically examined using the panel model, mediation effect model and spatial Durbin model. (1) This thesis can be seen in the research results; from 2011 to 2020, China’s DE maintained a stable growth trend in eastern, central and western regions. (2) Regardless of whether control variables are included or not, the DE can have a significant negative impact on CE and PCE at the 1% significance level, and every 1% increase in the DE reduces CE and PCE by 0.104% and 0.074%, suggesting that the DE protects the environment, which is proved by rigorous robustness and endogeneity tests. (3) The DE realises this result is caused by encouraging green technological innovation and consummating the industrial structure. Compared with industrial structure upgrading, DE has a more obvious effect on the number of green invention patents granted, and every 1% increase in DE increases the number of green invention patents granted by 0.857%. (4) Using two different spatial weight matrices and spatial Durbin models, this thesis can conclude that the DE can reduce local and neighbouring district CEs. (5) The following thesis can be founded by analysing the data at the regional level. The central and western regions have a greater ‘latecomer’s advantage’ in reducing carbon emissions, and for every 1% increase in the DE, the CE and PCE in the western region decrease by 0.194% and 0.084%. The DE is an economic development model that significantly influences CEs reduction. Sustainable development offers new solutions for global environmental management dilemmas.

Based on these findings, this study made the following recommended policy for developing countries.

1. The foundation of DE development should be consolidated. The authorities must raise funds for Internet infrastructure, amplify local digital infrastructure development capacity, hasten the pioneering application of Internet and other technologies and 5G in manufacturing firms, endeavour to create digital service platforms and establish avenues for information dissemination. This thesis must encourage businesses to utilise digital technology to rationally distribute, exploit and restructure their commodities and resources, bolster their efficiency and resource usage and facilitate achieving the ‘30–60’ dual carbon target via energy conservation and emission reduction.
2. The government should optimise CE reduction channels for the DE. National governments must focus on green technology innovation because it could accelerate the DE and reduce environmental pollution. Institute an inclusive, multi-faceted and equal-opportunity environmental policy framework, intensify financial backing for green technology research and development (R&D) initiatives, reinforce the leading position of firms in green technology innovation, enhance R&D potential for green technology innovation and accomplish a harmonised CEs reduction while promoting coordination of the two factors. Second, upgrading the industrial framework represents the most significant objective for China to stimulate sustainable development. China’s principal objective in fostering sustainable development is to maintain the DE as the lead determinant and advance the profound fusion of conventional and digital industries, invigorate the transformation of customary industries, particularly resource industries, to automation and environmental friendliness, utilise the digital platform to energise the role of digital technology in providing pertinent information for industrial growth and propel the transition of China’s industrial structure towards eco-friendliness and a low-carbon economy.
3. Play the radiation-driven effect of the DE. The governments must leverage cutting-edge digital technologies, including digitisation of production inputs, artificial intelligence and big data, to construct a framework for regional development that is synchronised, facilitates the cross-regional spread of technological innovation, reinforces positive spillover effects, activates technological demonstrations and knowledge transfer from adjacent areas, foster self-directed and interconnected development of the spatial economy, enhance integration with spatial technologies, encourage sound division of labour between different regions, minimise inter-regional developmental disparities and establish a self-sustaining cycle of economic growth and environmental stewardship throughout the region.
4. The government must consider regional disparities. Although different regions of China are major contributors to CEs, they lack adequate digital infrastructure, hindering their DE’s growth. When constructing a new platform for the DE’s expansion, the eastern region should prioritise technological innovation and take on a leadership role. This approach will enable China’s superior digital industries to permeate more quickly here, better integrate digital technology and traditional industry advantages and establish an all-inclusive green industrial chain to reduce CEs and improve environmental conservation.

It is important to acknowledge that this study primarily focuses on the macro-level influence of DE on CEs and its underlying mechanisms. Several limitations can guide future research. The DE encompasses diverse aspects, making it challenging to accurately measure its level of development using a single indicator system. The adoption of a more comprehensive indicator system becomes necessary. This study analyses the approach of the effect between DE development and CEs from both theoretical and empirical perspectives but fails to quantify the specific contribution of each mechanism. Future research could delve deeper into alternative impact mechanisms for advanced DE to affect CEs, employing quantitative assessments to identify the primary mechanisms at play. This paper addresses this issue at the macro level, lacking a micro-level explanation. With improved data availability, conducting more detailed micro-level analyses and utilising enterprise-level data would be valuable. This study exclusively explores the area level of how the DE affects CEs. Expanding research to investigate the influence of the DE on CEs in the inter-country heterogeneity represents a significant and worthwhile direction for future exploration.

## Supporting information

S1 File (XLSX)
